# Self-help cognitive behavioural therapy for hot flushes and night sweats during androgen deprivation therapy for prostate cancer: the MANCAN2 randomized controlled trial

**DOI:** 10.1038/s41416-026-03375-4

**Published:** 2026-03-24

**Authors:** Simon J. Crabb, Alannah Morgan, Evgenia Stefanopoulou, Louisa Fleure, Gareth O. Griffiths, Cherish Boxall, Sam Wilding, Theodora Nearchou, Sean Ewings, Jacqueline Nuttall, Zina Eminton, Emma Tilt, Emma Whitby, Bernard Siu, Paul Ridley, Lynsey Robson, Jenny Nobes, Joanne Preece, Roger Bacon, Jonathan Martin, Sarah Chamberlain, Deborah Fenlon, Myra Hunter, Alison Richardson

**Affiliations:** 1 Cancer Research UK Southampton Clinical Trials Unit, University of Southampton, Southampton, UK; 2 University Hospital Southampton NHS Foundation Trust, Southampton, UK; 3 Turning Point, London, UK; 4 Guys and St Thomas NHS Trust, London, UK; 5 Clatterbridge Cancer Centre, Merseyside, UK; 6 Royal Marsden NHS Foundation Trust, London, UK; 7 Ipswich Hospital, Ipswich, UK; 8 South Tyneside and Sunderland NHS Foundation Trust, Sunderland, UK; 9 Norfolk and Norwich University Hospitals NHS Foundation Trust, Norwich, UK; 10 Velindre Cancer Centre, Cardiff, UK; 11 PCaSO Prostate Cancer Support Organisation, Worthing, UK; 12 Department of Primary Care and Population Health, University College London, London, UK; 13 Faculty of Medicine, Health and Life Science, Swansea University, Swansea, UK; 14 Institute of Psychiatry, Psychology and Neuroscience, King’s College London, London, UK; 15 School of Health Sciences, University of Southampton, Southampton, UK

**Keywords:** Prostate cancer

## Abstract

**Background:**

Androgen deprivation therapy (ADT) causes hot flushes and night sweats (HFNS) and is associated with sleep disturbance, anxiety, low mood and cognitive impairment. We tested self-help cognitive behavioural therapy (CBT), when guided by prostate cancer nurse specialist teams, for mitigation of the long-term impact of HFNS, and associated symptoms.

**Methods:**

Prostate cancer patients receiving ADT, with a HFNS Problem Rating Scale ≥2, were randomised (1:1) to treatment as usual (TAU) or CBT + TAU, stratified by centre and treatment intent. CBT was a 4-week self-help intervention with pre- and post-intervention group workshops guided by trained prostate cancer nurse specialists. Primary endpoint: 6-month HFNS Problem Rating Scale. Secondary endpoints included HFNS frequency, ADT compliance and rating scales for HFNS beliefs and behaviours, quality of life, anxiety, depression and sleep.

**Results:**

162 patients were randomised. 6 month mean HFNS Problem Rating Scale score was not significantly different between the TAU and CBT + TAU groups (mean 4.08 vs 4.04, 95% confidence interval (CI) for difference: −0.89, 0.80; *p* = 0.97), although was improved at 6 weeks (mean 4.47 vs 3.79, 95% CI: −1.26, -0.09; *p* = 0.03), when depression, anxiety scores and ADT compliance also favoured CBT + TAU.

**Conclusions:**

The addition of CBT in prostate cancer patients receiving ADT improved short-term HFNS severity, in addition to improved anxiety and depression scores, but these were not maintained at 6 months.

**Clinical trial registration:**

ISRCTN58720120.

## Background

Prostate cancer (PCa) is the second most common cancer to affect men worldwide with 1.5 million new cases reported globally in 2022 [1]. Incidence and prevalence have continued to increase over the past 3 decades, predominantly through improvements in methods for detection. In contrast to many other cancers, overall PCa survival rates are high [2]. However, the incidence of clinically significant treatment-related side effects is also high with potential impact on quality of life for those living with, and beyond, a PCa diagnosis.

Up to 50% of patients diagnosed with PCa will receive hormonal therapy, comprising ADT, at some point during their treatment pathway, with intensification for some through combination with an androgen receptor targeted agent (ARTA) [3]. HFNS is the most common ADT side effect, occurring in up to 80% of patients and persisting in over half at five years [3, 4]. Furthermore, HFNS are associated with sleep disturbance, anxiety, depression, a negative impact on quality of life (QoL) and potentially ADT compliance [5, 6].

We have previously reported a prior, single-centre randomised study (MANCAN) which found that self-help CBT reduced the impact of HFNS due to ADT at an early time point of 6 weeks [7]. Of note, CBT was delivered by a clinical psychologist in this study (an assessment interview and one telephone session 2 weeks later), with potential implications for implementation of findings into routine clinical practice. In breast cancer patients, we have demonstrated that CBT delivered by breast cancer nurse specialists (CNS) in a randomised controlled trial across six UK hospitals improved HFNS impact at a later time point of 26 weeks (reduction in the mean HFNS problem rating scale of 46% with CBT compared to 15% with TAU, adjusted mean difference −1.96, 95% CI − 3.68 to −0.23, p = 0.039). In addition, this study detected improvements in sleep, anxiety and depression scores [8].

We hypothesised that the addition of self-help CBT, to standard approaches for the management of HFNS, would reduce their impact at longer time points in men with PCa experiencing problematic HFNS symptoms during ADT. Furthermore, we sought to evaluate whether this intervention could be effectively implemented and embedded into routine practice by prostate CNS teams.

## Methods

### Study design and patients

As previously described, MANCAN2 was an investigator-led, multicentre, randomised, clinical trial [9]. Patient eligibility criteria (Data Supplement) included a diagnosis of localised or advanced PCa. All patients were receiving ADT as part of their treatment programme with a plan for a minimum of 6 months further continuous treatment at the point of registration. ADT may have been planned to continue for either a fixed duration (for example following radiotherapy), or permanently, and administered with either adjuvant or palliative intent. It could be delivered pharmacologically, according to local institutional practice, or through surgical castration. Androgen receptor antagonists, abiraterone acetate, denosumab and bisphosphonates were all permitted, in combination with ADT, according to local institutional practice. All patients were experiencing problematic HFNS symptoms defined as a HFNS Problem Rating Scale score ≥2 [7]. This was calculated as the mean of three 10-point Likert scale scores assessing the extent to which HFNS are problematic, distressing and causing interference in daily life.

Patients had to be able to attend group workshops either through video conferencing software, in person or by telephone. Exclusion criteria included intermittent or neoadjuvant use of ADT, current evidence of disease progression or relapse, current chemotherapy, radical multi-fraction external beam radiotherapy or brachytherapy. Interventions intended to mitigate HFNS, including but not limited to, pharmacotherapy, herbal remedies, vitamin supplements, yoga and acupuncture were permitted according to local institutional practice.

### Procedures

Recruitment was conducted in hospitals throughout England and Wales. Each study site continued recruitment until a group of approximately 16 eligible participants was formed at that site. Eligibility was reconfirmed, and each participant then completed baseline questionnaires. The group was then randomly allocated, by computer-generated randomisation sequence, on a 1:1 basis, to either CBT + TAU, or TAU alone, stratified, with fixed block size, by recruiting centre and treatment intent (palliative or curative).

To allow for practical aspects of group randomisation in this manner, smaller or larger groups were permitted, as required, and some groups were amalgamated across trial sites. Although neither the participants nor the prostate CNS team were blinded to treatment allocation, the research team member collecting the 6-week and 6-month outcome data by telephone remained blinded throughout the trial as well as the researcher performing the statistical analyses.

All participants, regardless of randomised allocation, received TAU for HFNS symptoms as determined by their local care team and institutional practice. TAU was defined as care consistent with the National Institute for Health and Care Excellence (NICE) guideline NG131, Prostate cancer: diagnosis and management (https://www.nice.org.uk/guidance/ng131).

Men in the CBT + TAU intervention arm also received a 4-week self-help treatment schedule. The intervention content comprised an instructional self-help booklet (electronic or paper format) including information and exercises addressing stress management, paced breathing and cognitive/behavioural strategies to improve wellbeing and for managing hot flushes, night sweats and sleep, and a downloadable audio/CD demonstrating breathing/relaxation exercises. To further support patient’s engagement with the self-help booklet, participants engaged in two group workshops approximately 4 weeks apart facilitated by the prostate cancer CNS team at that site. The first offered practical help on how to use the self-help CBT guide appropriately and provided participants with an opportunity to meet other men experiencing similar symptoms. The second workshop focused on encouraging men to offer and receive emotional and practical support (peer support) in maintaining their gains in the future. Workshop delivery was either virtual through, video conferencing software or telephone, or in a face-to-face group setting with individual sites able to select which was utilised. Virtual training of the prostate CNS team to deliver workshops was conducted by a study clinical psychologist (ES). Team members had ongoing access to the training slides as well as a training manual to help support treatment fidelity during the trial. All group sessions were audio-recorded (with consent) and randomly selected (by computer-generated random number sequence) for one to two sessions per site to undergo fidelity analysis. An independent clinical psychologist (not involved in the CNS training) rated each selected workshop for delivery from the prostate CNS team for fidelity and adherence to the manual.

### Outcomes

Trial assessments (questionnaires) were each undertaken immediately prior to randomisation and then at 6 weeks and 6 months. These comprised the HFNS Problem Rating Scale, HFNS frequency (a subscale of the HFNS Rating Scale), the Hot Flush Beliefs and Behaviour Subscale for Men (HFBBS-Men), QoL (EORTC QLQ-C30), the Generalised Anxiety Disorder Questionnaire (GAD-7), depression through the Patient Health Questionnaire-9 (PHQ-9), functional impairment through the Work and Social Adjustment Scale (WSAS), sleep through the Pittsburgh Sleep Quality Index item 6 (PSQI) and ADT compliance (percentage of men compliant with the planned duration of ADT) [10–15]. In addition, we recorded the method of CBT workshop delivery (in person, virtual or hybrid), adverse events and CBT group workshop attendance.

MANCAN2 also included a process evaluation to investigate prostate CNS team experiences of delivering the intervention, a cost-effectiveness evaluation and a methodological study within a trial (SWAT) to understand if a theoretically informed cover letter could improve 6-month response rates to a postal questionnaire, which will be reported separately.

### Statistical analysis

Statistical analyses were pre-specified within an a priori statistical analysis plan. The primary endpoint was the difference in HFNS Problem Rating Scale, compared between the CBT + TAU and the TAU alone arms, measured at 6 months post randomisation. This endpoint was chosen because it (rather than HFNS frequency) is associated with help-seeking and quality of life, and, in recent studies, it has been recommended as a key patient-reported outcome measure in clinical trials of HFNS treatments [16, 17].

This was analysed using a linear mixed model, adjusted for baseline HFNS Problem Rating Scale, the stated treatment intent for ADT (curative or palliative) and centre. The trial was designed to detect a ≥ 1.5 point difference, between arms, at 6 months in the mean HFNS Problem Rating Scale which has been considered to be a clinically relevant difference [7, 8, 18]. This required 94 patients with a standard deviation (SD) of 2.21, 90% power and a 5% type 1 error. The sample size was then increased to 111 to account for clustering by intervention and intra-class correlation of 0.01 and further increased to 150 (75/arm) to allow for a 26% loss to follow-up, at the 6 month data collection point, based on our prior experience in breast cancer [8].

## Results

### Patients and treatment

**Fig. 1:**
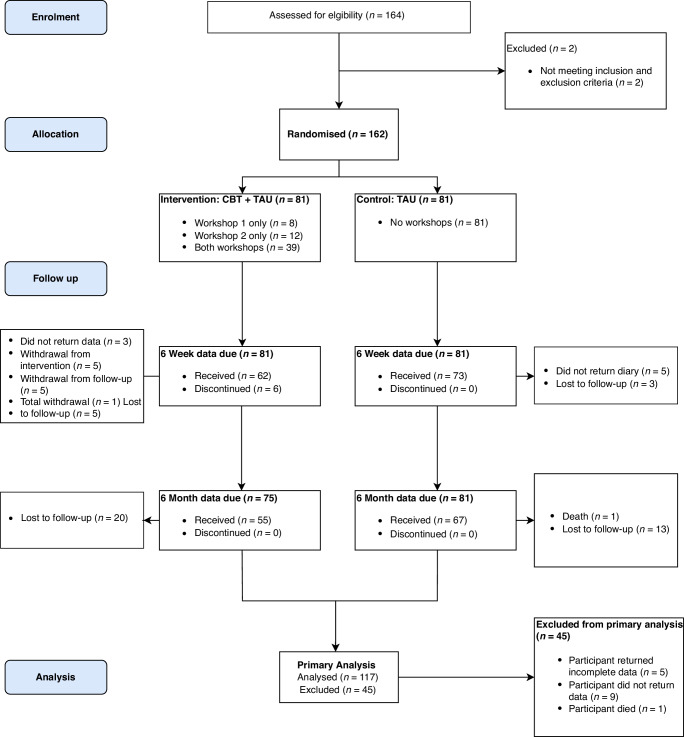
CONSORT diagram. TAU treatment as usual, CBT cognitive behavioural therapy.

164 patients were screened for eligibility between March 2022 and March 2023 (Fig. 1). 162 patients from nine cancer centres in the UK were randomly assigned (81 per arm) to either self-help CBT + TAU or TAU. Patient characteristics, at baseline, and for those returning data at 6 months, are summarised in Table 1 and were generally well balanced between groups. Median age of participants was 70 years (range 47-85). A majority of participants identified as having white ethnicity and UK origin (91.4%), were married or living with a partner (78.4%) and retired (69.8%). A larger proportion of patients were receiving ADT with palliative intent (61.7% and 63.0% in TAU and CBT + TAU groups, respectively), around half were taking a concurrent ARTA (51.9% and 49.4% in the TAU and CBT + TAU groups, respectively). Approximately 12% were receiving concurrent pharmacological or non-pharmacological interventions to attenuate HFNS.

**Table 1:** Patient characteristics at baseline and at 6 months.

	Baseline				6 months			
	TAU (*n *= 81)		CBT + TAU (*n *= 81)		TAU (*n* = 65)		CBT + TAU (*n* = 52)	
Age, median (min, max)	71	[53, 85]	69	[47, 85]	71	[53, 85]	69	[52, 85]
Treatment intent Curative Palliative	30 51	(37.0%) (63.0%)	30 51	(37.0%) (63.0%)	28 37	(43.1%) (56.9%)	16 36	(30.8%) (69.2%)
Time on ADT <1 year ≥1 year	41 40	(50.6%) (49.4%)	30 51	(37.0%) (63.0%)	35 30	(53.8%) (46.2%)	21 31	(40.4%) (59.6%)
Co-treatments^a^ ARTA^b^ Bicalutamide Bisphosphonate	42 9 10	(51.9%) (11.1%) (12.3%)	40 10 7	(49.4%) (12.3%) (8.6%)	31 7 7	(47.7%) (10.8%) (10.8%)	27 6 5	(51.9% (11.5%) (9.6%)
HFNS treatment None Drug based Non-drug-based	72 6 3	(88.9%) (7.4%) (3.7%)	71 5 5	(87.7%) (6.2%) (6.2%)	57 5 3	(87.7%) (7.7%) (4.6%)	45 5 2	(86.5%) (9.6%) (3.8%)
Observation period started January to August September to December	36 45	(44.4%) (55.6%)	37 44	(45.7%) (54.3%)	31 34	(47.7%) (52.3%)	23 29	(44.2%) (55.8%)
CBT workshop attendance No One Both			22 20 39	(27.2%) (24.7%) (48.1%)			7 13 32	(13.5%) (25.0%) (61.5%)

*TAU* treatment as usual, *CBT* cognitive behavioural therapy, *ADT* androgen deprivation therapy, *LHRH* luteinizing hormone-releasing hormone, *ARTA* androgen receptor targeted agent, *HFNS* hot flushes and night sweats.

^a^Options here are not mutually exclusive.

^b^included abiraterone acetate and prednisolone, enzalutamide, darolutamide, apalutamide.

At baseline, participants reported a median frequency of HFNS (sum of both hot flushes and night sweats) in the past week of 58 (inter-quartile range 42) across both treatment groups. The mean HFNS Problem Rating Scale score at baseline was 4.92 (SD = 2.42) in the TAU group and 5.04 (SD = 2.23) in the CBT + TAU group. Likewise, baseline scores for depression, anxiety, functional impairment, sleep quality and QoL were balanced by arm (Table 2). 52 patients (64.2%) receiving CBT + TAU and 65 patients (80.2%) receiving TAU provided complete data for the HFNS Problem Rating Scale score at baseline and 6 months, representing a 26.3% loss and consistent with our prior experience in breast cancer [8].

**Table 2:** Symptom scores at baseline.

Characteristic	CBT + TAU (*n *= 81)	TAU (*n* = 81)	Total (*n* = 162)
Patient Health Questionnaire-9 (PHQ9)			
Mean (SD^1^)	7.5 (5.89)	7.6 (6.25)	7.5 (6.05)
Median (IQR^1^)	6.5 (3.3–10.8)	6.0 (3.0–11)	6.0 (3.0–11.0)
Range	0–25	0–26	0–26
Missing, *n* (%)	3 (3.7%)	3 (3.7%)	6 (3.7%)
Generalised Anxiety Disorder Questionnaire (GAD-7)			
Mean (SD^1^)	5.7 (5.36)	4.7 (4.67)	5.2 (5.04)
Median (IQR^1^)	5.0(1.0–9.0)	3.0 (0–7.5)	4.0 (1.0–9.0)
Range	0–21	0–16	0–21
Missing, *n* (%)	1 (1.2%)	2 (2.5%)	3 (1.9%)
Work and Social Adjustment Scale Questionnaire (WSAS)			
Mean (SD^1^)	9.4 (8.84)	10.9 (10.77)	10.3 (10.0)
Median (IQR^1^)	9.0 (2.0–17.0)	8.0 (2.0–16.0)	8.0 (1.0–16.5)
Range	0–28	0–34	0–34
Missing, *n* (%)	44 (54.3%)	27 (33.3%)	71 (43.8%)
Sleep quality overall during the past month (PSQI) (0 = very good, 3 = very bad)			
Mean (SD)	2.7 (0.77)	2.7 (0.84)	2.7 (0.81)
Median (IQR)	3.0 (2.0–3.0)	3.0 (2.0–3.0)	3.0 (2.0–3.0)
Range	1–4	1–4	1–4
Missing, *n* (%)	2 (2.5%)	0 (0.0%)	2 (1.2%)
QoL Questionnaire (EORTC QLQ-C30)			
Mean (SD)	59.6 (19.45)	62.0 (21.61)	60.8 (20.54)
Median (IQR)	58.3 (50.0–75.0)	66.7 (50.0–75.0)	66.7 (50.0–75.0)
Range	16.7–91.7	0–100	0–100
Missing, *n* (%)	1 (1.2%)	0 (0.0%)	1 (0.6%)

The majority of workshops delivered to patients in the CBT + TAU arm were either in-person or hybrid (in-person and virtual) sessions (61.7% and 22.2% respectively). 22 of 24 workshop sessions were audio recorded, of which 16 (73%) were randomly selected for assessment of fidelity and adherence to the manual. On average, 7/8 session aims from each reviewed workshop were considered adequately covered by independent review by an independent clinical psychologist.

### HFNS Problem Rating Scale

**Fig. 2:**
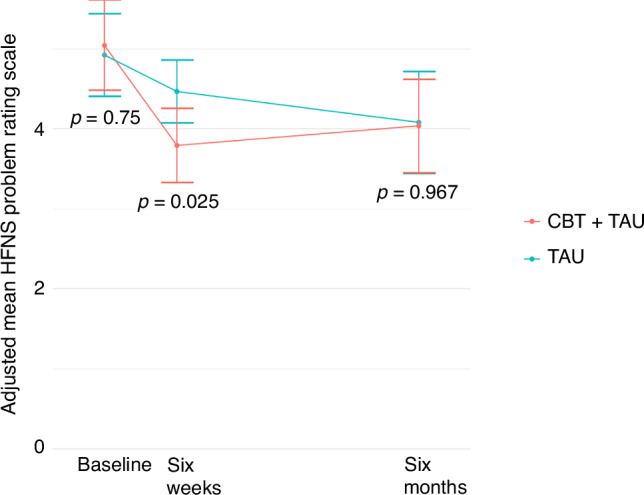
HFNS Problem Rating Scale scores. Changes in HNFS Problem Rating Scale score in CBT + TAU and TAU groups from baseline to 6 weeks and 6 months post randomisation. Error bars represent the 95% CI. HFNS hot flushes and night sweats, CBT cognitive behavioural therapy, TAU treatment as usual.

At 6 months, no difference was detected in the mean HFNS Problem Rating Scale score between the CBT + TAU and TAU groups. Mean scores at 6 months were 4.04 and 4.08 in the CBT + TAU and TAU groups respectively (95% CI -0.829 to 0.916; *p* = 0.967; Fig. 2).

A statistically significant difference in mean HFNS Problem Rating Scale score was detected between treatment groups at 6 weeks, with a greater reduction from baseline in the CBT + TAU group compared to the TAU group (mean 4.47 vs 3.79, 95% CI: −1.267 to −0.085; *p* = 0.025; Fig. 2).

This pattern (benefit at 6 weeks but not maintained at 6 months) remained true for subgroup analyses (Supplementary Figs. 1 and 2).

### Secondary end points

**Fig. 3:**
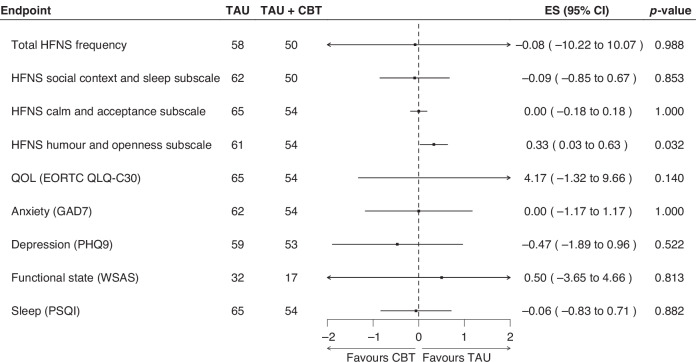
Forest plot of secondary endpoints at 6 months. TAU treatment as usual, CBT cognitive behavioural therapy, ES effect size, CI confidence interval, HFNS hot flushes and night sweats, QOL quality of life.

As with the HFNS Problem Rating Scale score, we did not see a difference in any of the specified secondary endpoints at 6 months (Fig. 3).

**Fig. 4:**
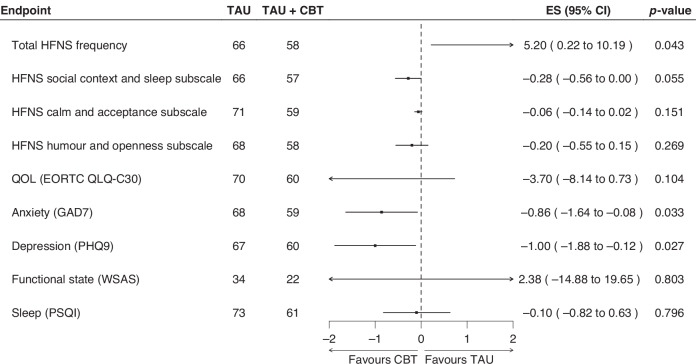
Forest plot of secondary endpoints at 6 weeks. TAU treatment as usual, CBT cognitive behavioural therapy, ES effect size, CI confidence interval, HFNS hot flushes and night sweats, QOL quality of life.

However, we did identify a statistically significant improvement in scores for patient anxiety (GAD-7; adjusted mean difference 0.857, 95% CI 0.079 to 1.635; *p* = 0.033) and depression (PHQ9; adjusted mean difference 1.00, 95% CI 0.124–1.876; *p* = 0.027) which favoured CBT + TAU at 6 weeks (Fig. 4). At 6 weeks, CBT + TAU was also associated with a higher HFNS frequency than TAU (weekly median 54.2 vs 59.4, 95% CI 0.22 to 10.19; *p* = 0.04). Differences in HFNS frequency between arms appeared sensitive to assumptions about missing data.

No significant differences were identified for HFNS Beliefs and Behaviours subscales (social context and sleep, calm and acceptance, and humour and openness), QoL (EORTC QLQ-C30), functional impairment (WSAS) or sleep (PSQI) between the treatment groups at either time point.

ADT compliance appeared to favour the CBT + TAU arm with 9 of 65 (13.8%) of control arm patients randomised to TAU discontinuing ADT by 6 months compared to none of the 51 patients in the CBT + TAU arm (*p* = 0.006). There were no adverse events reported in the CBT + TAU arm and one in the TAU arm resulting from a fatal myocardial infarction (deemed unrelated to TAU).

## Discussion

The MANCAN2 clinical trial did not demonstrate improvement in HFNS impact, or associated symptoms, at 6 months as a result of a 4-week self-help CBT programme during ADT for PCa. This was despite seeing reduction in the impact of HFNS, in addition to improved anxiety and depression scores, at 6 weeks in those that received CBT. This initial benefit appeared consistent across clinically important subdivisions, including treatment intent (curative or palliative), prior duration of hormonal therapy and use of hormonal therapy intensification (with ARTA drugs).

Several factors may have influenced these findings. There is a tendency for HFNS impact to decline over time and this was observed in our control arm. One interpretation of our results is that the addition of CBT mitigated HFNS impact earlier than would otherwise be the case. However, at six weeks the benefit of incorporating CBT with TAU may partly be due to a non-specific effect of attending two workshops relatively recently and having more individualised attention from healthcare professionals. Patient motivation and engagement is fundamental to the success of self-help interventions. We might anticipate that some patients experience fatigue from CBT-based ‘work’ which reduced their engagement and therefore the efficacy of this intervention at a later time point. Or that specific components are in some way a barrier to CBT success in the long term, such as reading, or comprehending, the instruction booklet or motivation for undertaking stress reduction exercises by oneself. Additional patient support from a prostate CNS and wider healthcare teams to remind and motivate patients beyond the initial 4-week CBT programme might potentially address this and could be the subject of future investigation. CBT workshop attendance at both workshops was 48.1% in those assigned to CBT + TAU and 61.5% in those returning data at 6 months. The reasons were not explored in this trial but identifying and addressing barriers to attendance could help increase rates and potentially improve longer term CBT engagement.

Our prior work in breast cancer is not exactly comparable in terms of the design of the CBT schedule (6 sessions at weekly intervals). In that experience, 73.8% of participants allocated to receive CBT attended at least 4 sessions [8]. The optimal frequency and duration of support to patients for CBT remains uncertain and, as we attempted to address in design of this study, needs to accommodate realistic expectations to enable implementation. In addition to gender, other factors that may have influenced the differences seen in this study, compared to our prior work in breast cancer, may have included age (median 71 for MANCAN2 compared to 54) and as a result of a greater likelihood of co-morbidities.

In this study, training of existing prostate CNS teams to support the implementation of self-help CBT was found to be viable and supports previous results in breast cancer [8]. Delivery by a clinical psychologist does not, therefore, appear to be necessary following provision of adequate training. This has important implications when considering how interventions for HFNS could be effectively incorporated into existing pathways of care. A nurse-led solution represents a pragmatic approach to the potential challenges in implementing self-guided psychosocial interventions for cancer patients, with the benefit of utilising existing healthcare professionals. And indeed it may also benefit from the fact that patients are likely to have an established rapport.

Interestingly, 13.8% of control arm patients discontinued ADT by 6 months compared to none in the intervention arm. The reasons for this were not directly evaluated in this study. It is therefore uncertain whether this observed effect may be directly related to HFNS symptoms and their impact, associated factors such as the early improvement in anxiety and depression scores, or other factors such as positive impact through increased contact with healthcare professionals. Further research should now investigate the potential role for CBT to support treatment compliance.

At present, there are few effective treatment options to manage HFNS symptoms during ADT. Pharmacological approaches are of limited efficacy with a risk of side effects. A systematic review of treatments for HFNS in PCa concluded that diethylstilbesterol, megestrol acetate and medroxyprogesterone are the most effective treatments, but all have side effects that impact tolerance [19]. The most robust prospective study, randomised 311 men with established troublesome hot flushes after 6 months on ADT, and found cyproterone and medroxyprogesterone to be more effective than venlafaxine [20]. However, the lack of a control arm, and the tendency for a reduction in HFNS over time, limit the conclusions that may be drawn. Current guidance from the National Institute for Health and Care Excellence (NICE) in the UK recommends offering medroxyprogesterone to manage troublesome hot flushes caused by long-term androgen suppression and to consider cyproterone acetate if this is ineffective or poorly tolerated.

Non-pharmacological mitigation options to address ADT-induced HFNS lack adequate prospective data [9]. There is, however, a growing body of evidence elsewhere to support the use of CBT as a safe and effective intervention to help manage some of the negative impacts of HFNS and to improve psychosocial function. A 2020 literature review, including 12 studies evaluating non-pharmacological approaches for the management of menopausal vasomotor symptoms in breast cancer patients suggested that current evidence supports CBT and hypnosis as potential treatment options [21]. There is evidence that structured CBT, focusing on key elements of the experience of HFNS, is effective for the alleviation of symptoms in both women with, and without, breast cancer [22].

The use of pharmacological and non-pharmacological options for mitigation of HFNS in this trial was low at 12% (and this could be viewed as a limitation). This likely reflects real-world circumstances, at least in the UK, as a result of clinician views on efficacy and patients wishing to avoid further medication (with their own side effect profile) to address side effects from hormonal therapy. We chose not to mandate pharmacological intervention in this trial to reflect the reality of current practice. Where they were utilised, we did not detect any impact of such approaches on the HFNS Problem Rating Scale in subgroup analyses at either 6 weeks or 6 months (Supplementary Figs. 1 and 2). However, as they were only used in 12%, this should be interpreted with some caution due to the small numbers involved.

Potential limitations of MANCAN2 included the inability to mask participants to their treatment allocation. This study was also not designed to evaluate patient adherence to CBT techniques in response to HFNS following completion of the 4-week programme. In addition, our data raise the question of whether the intensity of CBT facilitation by CNSs, or duration beyond 4 weeks, are drivers for long-term durability of effect.

In conclusion, we have confirmed, in a multicentre setting, that CBT in men on ADT is effective at reducing troublesome HFNS impact at 6 weeks [7]. Furthermore, it can be facilitated successfully by the existing prostate CNS team. However, the impact on HFNS was not sustained at 6 months. The importance of multifaceted interventions to address the psychosocial impact of hormonal interventions for PCa patients is well documented [23]. Further research should address the durability of the impact of self-help CBT, how one might support patients to maintain progress evidenced at earlier time points and explore how CBT might be effectively incorporated into a comprehensive therapeutic approach.

## Supplementary information

Supplementary Figure 1 and 2

## Data Availability

The authors are committed to the responsible sharing of clinical trial data with the wider research community. To access or share this trial data set please contact the SCTU Data Release Committee through ctuadmin@soton.ac.uk or the corresponding author.
