# Supplementary material for: Self-help cognitive behavioural therapy for hot flushes and night sweats during androgen deprivation therapy for prostate cancer: the MANCAN2 randomized controlled trial
Source: Br J Cancer. 2026 Mar 24;134(10):1413–9. doi: 10.1038/s41416-026-03375-4 (PMC13133258; doi:10.1038/s41416-026-03375-4)

**Data Supplement**

Self-help cognitive behavioural therapy for hot flushes and night sweats during androgen deprivation therapy for prostate cancer: the MANCAN2 randomized controlled trial

Simon J. Crabb, et al.

**Supplementary Figure 1.** Forest plot for subgroup analysis of the mean HFNS Problem Rating Scale at month 6 (modified ITT population, n=117)

Hormone therapy at baseline relates to use whether patients were receiving an androgen receptor targeted agent in addition to androgen deprivation therapy or not.

*Due to zero cell counts, these p-values are in comparison to the TAU population not attending any workshops.

TAU, treatment as usual; CBT, cognitive behavioural therapy; ES, effect size; CI, confidence interval; HFNS, hot flushes and night sweats


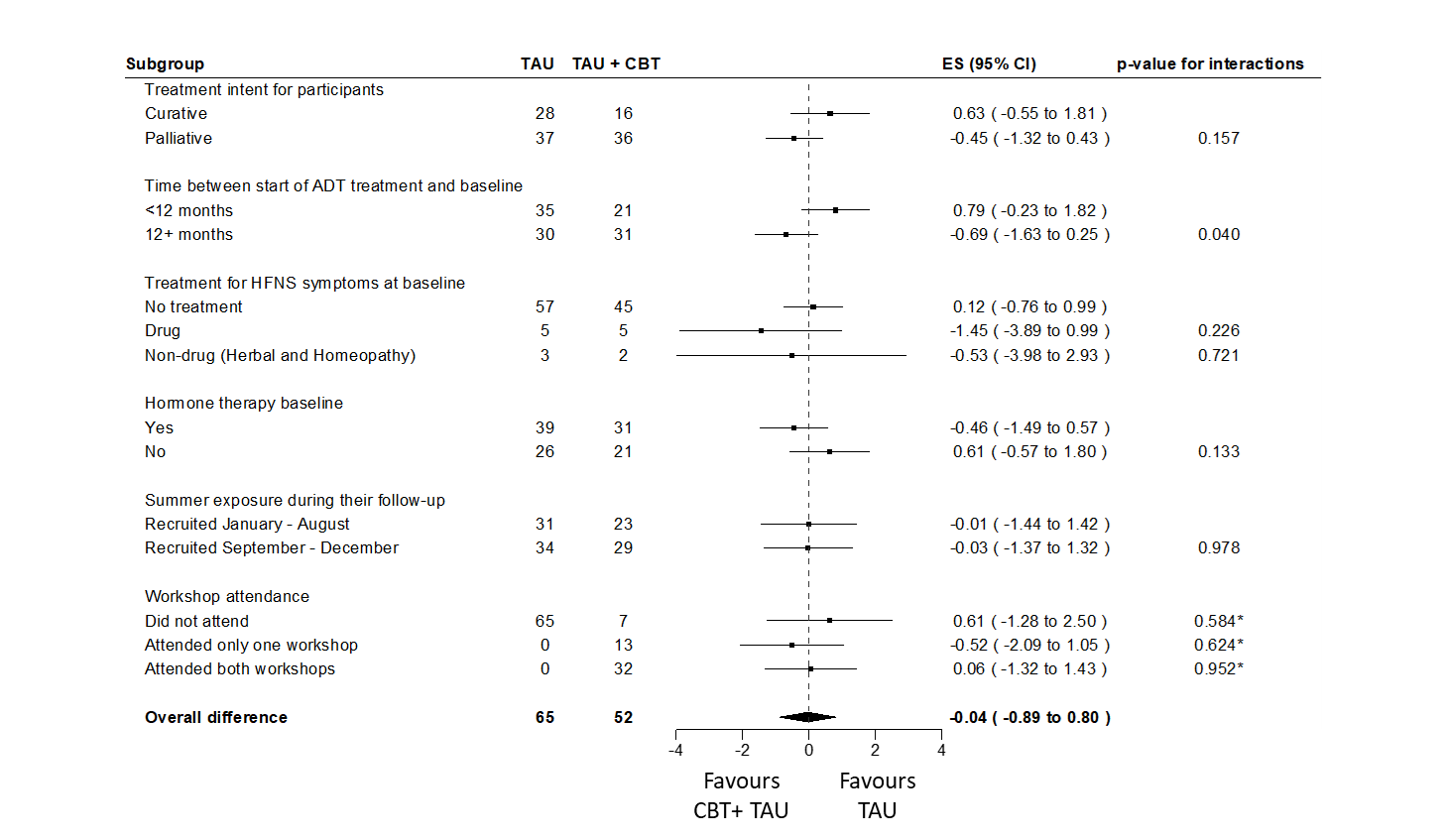


**Supplementary Figure 2.** Forest plot for subgroup analysis of the mean HFNS Problem Rating Scale at week 6 (modified ITT population, n=131)

Hormone therapy at baseline relates to use whether patients were receiving an androgen receptor targeted agent in addition to androgen deprivation therapy or not.

*Due to zero cell counts, these p-values are in comparison to the TAU population not attending any workshops.

TAU, treatment as usual; CBT, cognitive behavioural therapy; ES, effect size; CI, confidence interval; HFNS, hot flushes and night sweats


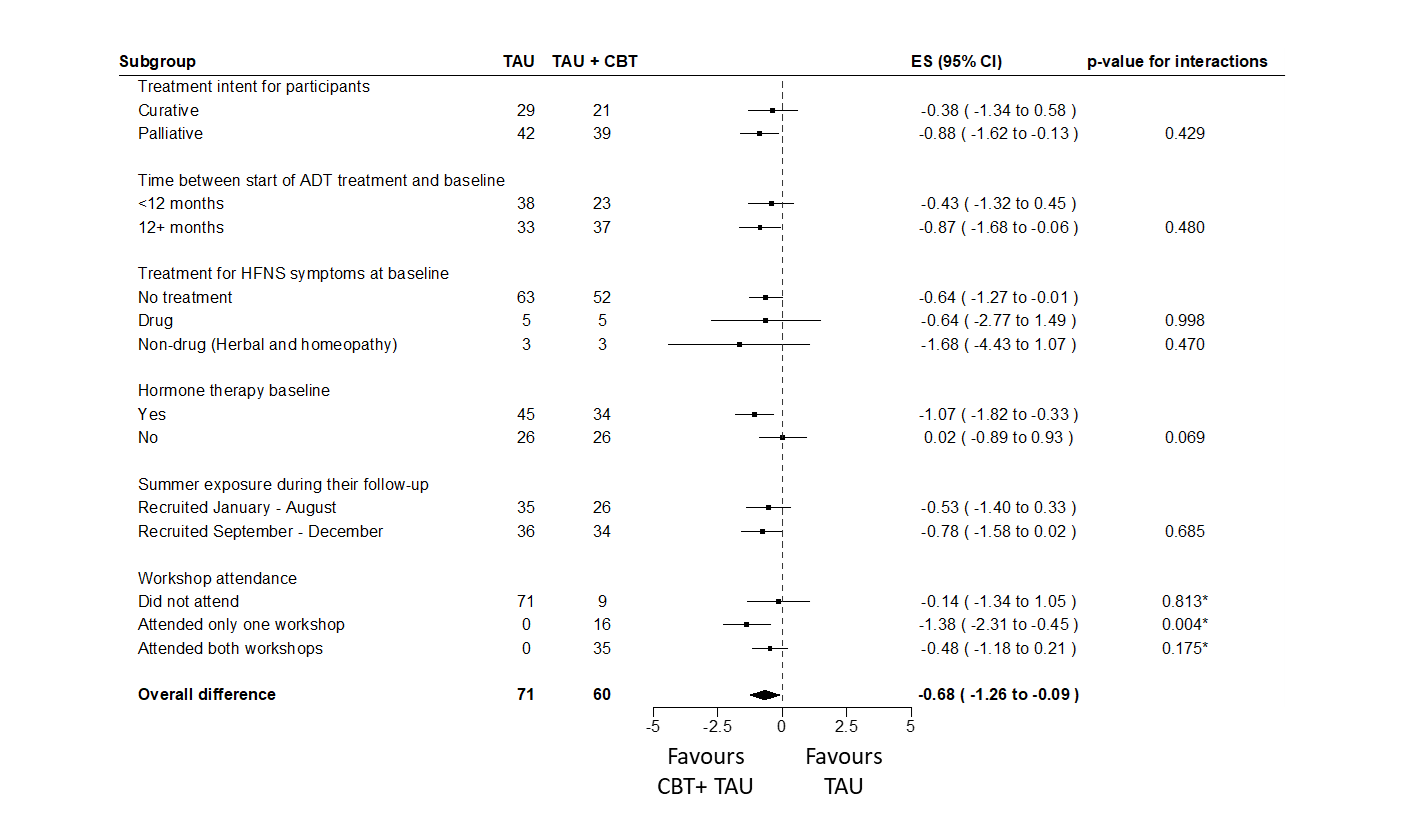

Supplement: Supplementary file 1 — Supplementary Figure 1 and 2 [file 41416_2026_3375_MOESM1_ESM.docx]
